# The Latest Treatment for Head and Neck Cancers: Transoral Robotic Surgery and Photoimmunotherapy

**DOI:** 10.14789/jmj.JMJ22-0038-R

**Published:** 2023-03-13

**Authors:** FUMIHIKO MATSUMOTO

**Affiliations:** 1 Department of Otorhinolaryngology, Faculty of Medicine, Juntendo University, Tokyo, Japan; 1 Department of Otorhinolaryngology, Faculty of Medicine, Juntendo University, Tokyo, Japan

**Keywords:** head and neck cancer, trans oral robotic surgery, photo immune therapy

## Abstract

Remarkable progress has been achieved in head and neck surgery in recent years. Transoral robotic surgery (TORS) is becoming popular worldwide for the removal of less invasive tumors. TORS is used especially for oropharyngeal cancer and is minimally invasive, with a short treatment time and minimal postoperative dysfunction. The procedure is performed by inserting one endoscope and two robotic arms. The robotic arm has a 360-degree movable tip, which enables fine manipulation, even in narrow oral cavities. The endoscope also provides three-dimensional images and enables the surgeon to get close to the operative site, making it possible to check for small blood vessels and other objects while performing the operation.

Photoimmunotherapy is a new treatment for distant metastasis or recurrent head and neck cancers not indicated for surgery or radiotherapy. Treatment requires the administration of a dye, IR700, one day before the procedure. This dye disrupts cell membranes when exposed to near-infrared (NIR) radiation. To deliver this dye specifically to the tumor, an antibody drug against the epidermal growth factor receptor, which is expressed relatively specifically in tumors, is used. This treatment has a strong anti-tumor effect and the tumor shrinks relatively quickly. However, because NIR irradiation is required, the lesion must be within the infrared irradiation range. In addition, because of rapid shrinkage of the tumor, post-treatment tissue defects are serous complication, and tumor invasion into the carotid artery are contraindications due to the risk for major hemorrhage caused by tumor shrinkage.

## Introduction

The field of otorhinolaryngology is diverse and includes the disciplines of otology, rhinology, and head and neck surgery. Head and neck surgery addresses tumors that occur in the head and neck region and is a field that has witnessed remarkable progress in recent years. Radiotherapy and surgery using external incisions are conventional curative treatments for head and neck cancers. In addition, drug therapy is commonly used in cases of distant metastasis or recurrence, although it is difficult to achieve complete cure using drug therapy alone. In the present article, we introduce and discuss robotic surgery and photoimmunotherapy as the latest treatments for head and neck cancers.

## Transoral robotic surgery

Recent advances in endoscopic techniques, especially the introduction of narrowband imaging (NBI), have made it possible to detect pharyngeal cancer at an earlier stage than in the past. NBI is a pioneering technology developed in the field of gastrointestinal endoscopy that has been recently applied to the head and neck region. NBI is an optical imaging enhancement technology that depicts capillaries in the superficial layer of the mucosa in brown and those in the submucosal tissue in blue-green on a monitor, thus enabling identification of lesion (s) that develop from capillaries characteristic of superficial cancer.

Efforts are now being made to perform less-invasive resection of cancers detected at an early stage using these techniques, and robots can now be used to perform less-invasive resection (s). Cases that previously required external incisions or radiotherapy can now be treated less invasively and in a shorter period by removing the cancer transorally. The conventional external incision approach results in an incision wound on the neck, which is inferior in terms of cosmetic appearance. In addition, the neck wound heals with scarring, causing postoperative discomfort and neck contracture. In addition, postoperative swallowing function is impaired due to scar contracture and the resection of muscles necessary for swallowing. Radiotherapy is an alternative to the external incision approach; however, it is also associated with decreased quality of life after treatment due to scarring of cervical tissues caused by radiation injury, which results in impaired swallowing function and salivary secretion. However, when tumors are removed transorally using a robot, surgery can be performed without incisions in the neck, which is preferred in terms of esthetics, and contracture caused by manipulation (s) of the neck can be prevented. In addition, because surgical manipulation is limited to the area around the resection site, postoperative functional deterioration due to scar contracture and muscle dehiscence can be minimized. In actual clinical practice, patients can be discharged from hospital relatively early (approximately one week after surgery) and, although some decline in swallowing function may occur, it can be minimized^1, 2)^. A “big data” analysis by the United States National Cancer Database reported that the advantages of TORS included low rates of positive resection margins and postoperative chemoradiotherapy^3)^, and that TORS significantly prolonged overall survival compared with other modalities in human papilloma virus-positive oropharyngeal cancer^4)^. Sano et al. reported a lower rate of positive margins with TORS than with other transoral resection modalities such as transoral video surgery and endoscopic laryngo-pharyngeal surgery^5)^. Thus, diverse evidence supporting TORS has accumulated; however, not all head and neck cancers are indications for the procedure. Nevertheless, the lateral and anterior walls of oropharyngeal cancers are considered to be good indications. TORS has been widely used in the United States and Europe, and Japan was somewhat late in introducing robotic surgery to this area. However, insurance has covered TORS in Japan since April 2022, and the number of facilities capable of performing the procedure is gradually increasing.

**Figure 1:**
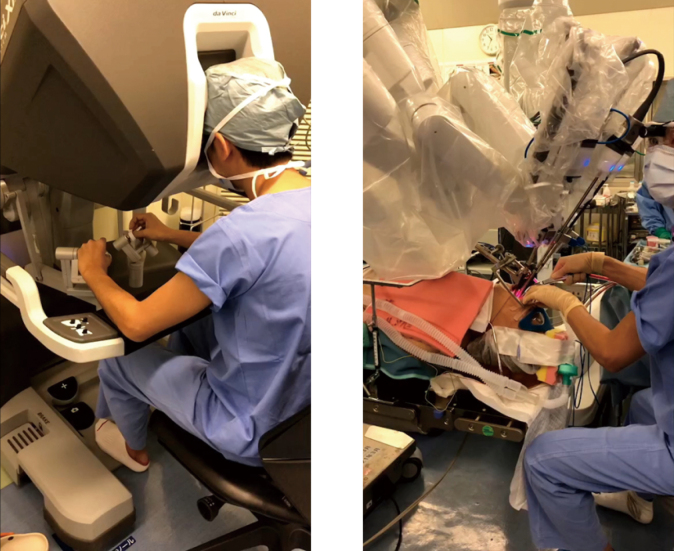
A: The surgeon operates remotely from a console. B: Three robotic arms are inserted orally to perform the operation. An assistant provides support near the patient.

TORS is performed by inserting a camera and two robotic arms (three in total) through the mouth. The robotic arm has a 360-degree movable tip, which enables detailed manipulation, even in the narrow oral cavity (Figure 1). The robotic arm is equipped with an anti-shake function such that, even if the surgeon's hands shake on the console, the robotic arm will not shake. The robot arm is also equipped with a scale function, so that the actual robotic arm moves only 1 cm when the surgeon moves his hand 5 cm; this scale can be set to 1:3 or 1:5. These features enable more precise manipulation. In addition, the endoscope provides high-quality three-dimensional images and affords the surgeon close access to the operative site, making it possible to safely proceed with the procedure while confirming the presence of small blood vessels. An important point when performing surgery is the development of a good surgical field using a mouth gag. Recently, new and improved apertures have been introduced to provide a better surgical environment. One disadvantage of robotic surgery is that the robotic arm does not have a sense of touch; as such, it is not possible to palpate the hardness of tissue, and the three arms are inserted into the narrow oral cavity; therefore, it is necessary to operate the arms carefully to avoid interference among them.

## Photoimmunotherapy

**Figure 2:**
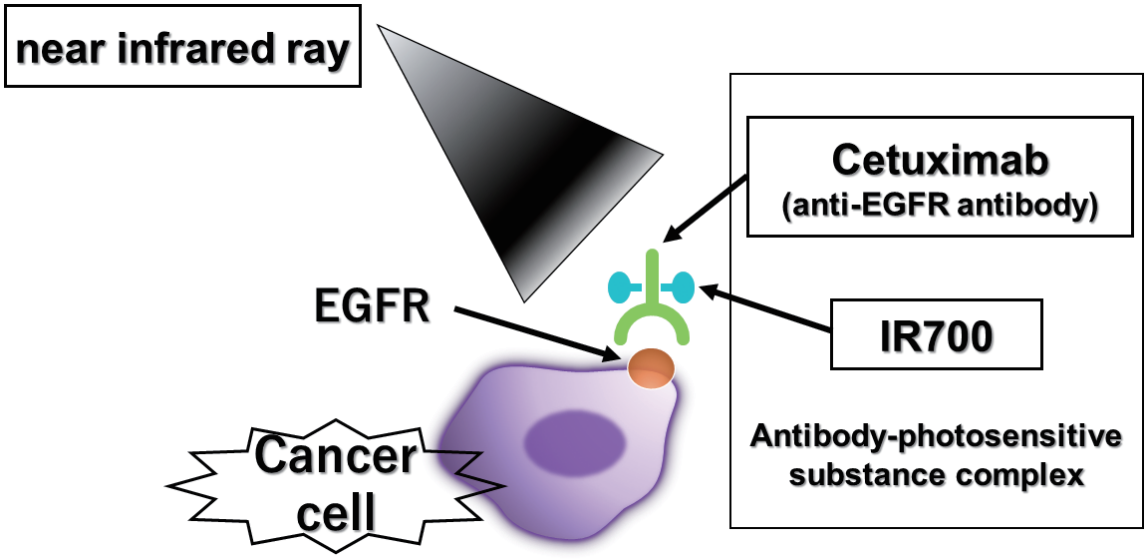
Mechanism of photoimmunotherapy

**Figure 3:**
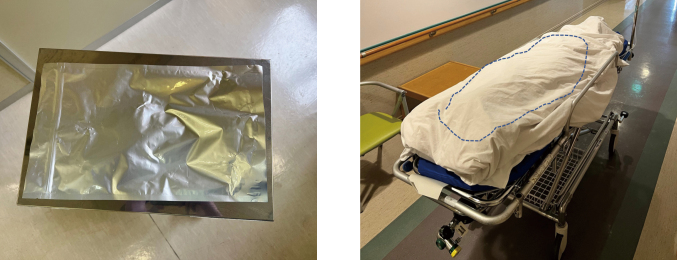
A: The drug needs to be covered with a shield to prevent exposure to light. B: The patient is transported to the operating room under a cover to prevent exposure to light.

**Figure 4:**
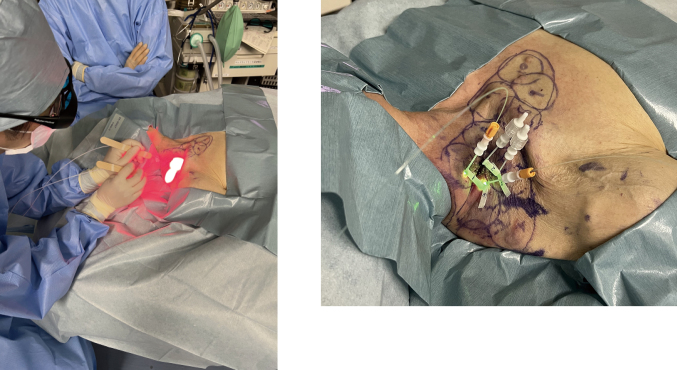
A: Frontal diffuser B: Cylindrical diffuser

Photoimmunotherapy is a new treatment method that was conditionally approved in Japan in September 2020. It is indicated only for head and neck cancers in patients with recurrent or metastatic disease and for whom none of the existing treatments, such as radiation or surgery, are indicated. The treatment mechanism is described as follows. A dye, IR700, which causes a chemical reaction when irradiated by near-infrared radiation (NIR), is used to damage cell membranes. This dye is administered intravenously to the patient before NIR irradiation. When this dye is exposed to light, it becomes insoluble and damages cell membranes, thereby destroying cancer cells. To make the treatment more effective, the dye must specifically bind to cancer cells and not to normal cells. To achieve this, cetuximab, an antibody against the epidermal growth factor receptor (EGFR), which is highly expressed in head and neck cancer cells, is combined with the dye and administered. This makes it possible to specifically target and bind the dye to cancer cells without binding to normal cells. Then, irradiation with NIR, which causes a reaction with the dye, causes the dye to bind to the surface of cancer cells, react, and destroy them^6-8)^ (Figure 2). The day before treatment, a drug containing IR700 combined with an anti-EGFR antibody is administered intravenously. Because the drug can react when exposed to light at this time, it is handled under a light shield. On the day of the procedure, the patient is transferred to the operating room for NIR irradiation of the tumor under general anesthesia; however, during this time, the dye will react if the patient is exposed to light. Therefore, the patient is transferred under a light shield (Figure 3) and the operating room is kept dark. There are two methods for irradiating lesions using NIR: frontal and cylindrical. A frontal diffuser is a method in which light is irradiated from the front as if shining a flashlight. The cylindrical diffuser is a method in which a needle is inserted into the tumor, and an optical fiber is inserted into the needle to emit light from the inside (Figure 4). Irradiation is performed by skillfully combining these two methods, considering the extent and thickness of the lesion. This therapy has a high antitumor effect on cancer cells, and the tumor often shrinks relatively quickly; however, tissue defects due to tumor shrinkage are a major problem. In addition, if the tumor has invaded the carotid artery, treatment is contraindicated due to the risk for major bleeding caused by tumor shrinkage. The conditions for this treatment are that the tumor must have a relatively specific target―such as EGFR―to enable the dye to bind to the tumor while bypassing normal areas, and that the tumor must be located in an area that can be irradiated with NIR. For these reasons, as mentioned above, head and neck cancer is a current indication for treatment^9)^. Research and development of new methods, such as the search for molecular targets to bind dyes specific to cancer cells in other types of cancers and NIR irradiation from the tip of an endoscope, are currently underway, and it is anticipated that this technology will be expanded to other areas in the future.

In addition to the direct destruction of tumors, this therapy also has other potential effects. More specifically, it enhances immunity against tumors. Conventional surgery, radiotherapy, and chemotherapy all exert anti-tumor effects; however, there is concern that the invasiveness of surgery and the side effects of treatment may reduce the physical strength of the patient. While photoimmunotherapy requires general anesthesia, the burden on the patient is much less than that of conventional therapies. Tumor cells are destroyed without damaging the surrounding tumor tissue; as such, undamaged tumor antigens are dispersed from the destroyed tumor cells into the body. Tumor antigens are recognized by dendritic cells and other cells, which potentially enhance the patient's own tumor immunity. In turn, this enhancement of tumor immunity may have an abscopal effect, reduce the size of untreated distant metastatic lesions and prevent recurrence after treatment. Therefore, the treatment is referred to as “photoimmunotherapy”. Currently, however, there are no clear data supporting such effects in clinical practice, although further evidence is anticipated to be accumulated in the future. Methods, such as combining therapy with existing immune checkpoint inhibitors, such as nivolumab and ipilimumab, to further enhance tumor immunity for cancer treatment, are being investigated. New developments are anticipated in the future, such as the use of this method in other therapies and other aspects of treatment^10)^.

## Conclusion

Various new treatments have been developed and introduced in the field of head and neck cancer. This has led to improved prognosis and minimally invasive treatment. Further advances and development are anticipated in the future.

## Funding

No funding was received.

## Author contributions

FM drafted the manuscript. FM read and approved the final manuscript

## Conflicts of interest statement

The author declares that there are no conflicts of interest.
